# Cholécystectomie laparoscopique sur *situs inversus*

**DOI:** 10.11604/pamj.2018.31.183.14061

**Published:** 2018-11-15

**Authors:** Rachid Boufettal, Driss Erguibi, Amal Hajri, Anass Idrissi, Saad Rifki Jai, Farid Chehab

**Affiliations:** 1 Service de Chirurgie Générale III, CHU Ibn Rochd, Casablanca, Maroc; 2 Faculté de Médecine et de Pharmacie Casablanca, Université Hassan II, Casablanca, Maroc

**Keywords:** écystectomie, laparoscopie, *situs inversus*, cardiopathie, laparoscopy, situs inversus, heart disease

## Abstract

La coeliochirurgie est la technique de référence pour le traitement de la lithiase vésiculaire symptomatique. Jusqu’à ce jour, seuls 42 cas de cholécystectomie laparoscopique chez des patients présentant un *situs inversus* ont été publiés. Ainsi, nous rapportons un nouveau cas d'un patient, suivi pour cardiopathie congénitale complexe à type de ventricule unique sur *situs inversus* et dextrocardie. C’est un patient qui était hospitalisé pour prise en charge chirurgicale de lithiase vésiculaire symptomatique. Une cholécystectomie laparoscopique était réalisée. La disposition des trocarts et l’abord chirurgical étaient complètement inverses et symétriques par rapport à l’abord laparoscopique normal.

## Abstract (en)

Coelio-surgery is the gold standard treatment for symptomatic gallbladder lithiasis. Until now, only 42 cases of laparoscopic cholecystectomy in patients with situs inversus have been published. We here report a new case of a patient followed up for complex congenital single ventricle heart disease with situs inversus and dextrocardia. The patient was admitted for the surgical treatment of symptomatic gallbladder lithiasis. Laparoscopic cholecystectomy was performed. Disposal of trocars and surgical approach were completely inverted and symmetric compared to standard laparoscopy.

## Introduction

Le *situs inversus* (SI) est une malformation congénitale rare. Elle est à l’origine de difficultés diagnostiques et thérapeutiques rencontrées dans de nombreuses situations cliniques, surtout si le malade n’est pas connu comme porteur de cette malformation [1]. C’est le cas de la lithiase vésiculaire sur SI. Nous rapportons une nouvelle observation de cholécystectomie laparoscopique pour lithiase vésiculaire symptomatique sur *situs inversus* complet avec cardiopathie. Nous discutons des difficultés diagnostiques et des techniques de l'abord laparoscopique inhérentes à cette anatomie en miroir.

## Patient et observation

**Figure 1:**
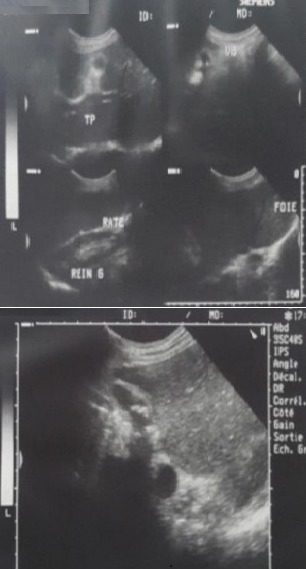
échographie abdominale, réalisée chez notre patient, montrant une lithiase vésiculaire sur SI

**Figure 2:**
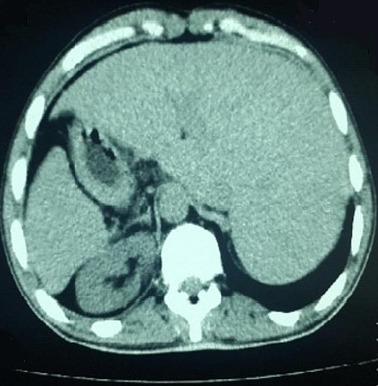
TDM abdominale réalisée chez notre patient montrant le SI

Il s’agit d’un patient âgé de 25 ans, suivi pour cardiopathie congénitale complexe à type de ventricule unique, *situs inversus* et dextrocardie. Il a été opéré à l’âge de 18 ans pour dérivation bicavopulmonaire avec une bonne évolution clinique et mis sous cardioaspirine. Par ailleurs, le patient avait présenté 2 mois avant son admission, une douleur de l’hypochondre gauche à type de colique hépatique. A l’admission le patient était apyrétique et anictérique. L’abdomen était souple à la palpation et le toucher rectal était sans particularité. Par ailleurs, l’examen pleuropulmonaire avait objectivé la présence d’une cicatrice de sternotomie, alors que le choc de pointe était à droite à l’examen cardiovasculaire. L’échographie abdominale avait objectivé une lithiase vésiculaire sur SI avec un épaississement de sa paroi. Les voies biliaires intra et extra hépatique étaient de calibre normal. Le reste de l’exploration était sans particularité (Figure 1). Le bilan hépatique était normal. Un scanner abdominal était demandé (Figure 2), confirmant le *situs inversus*, la vésicule biliaire était le siège de quelques calculs infra centimétriques, un petit kyste médio rénal droit parenchymateux centimétrique, sans autres anomalies décelables notamment absence de dilatation des voies biliaires intra et extra hépatiques.

**Figure 3:**
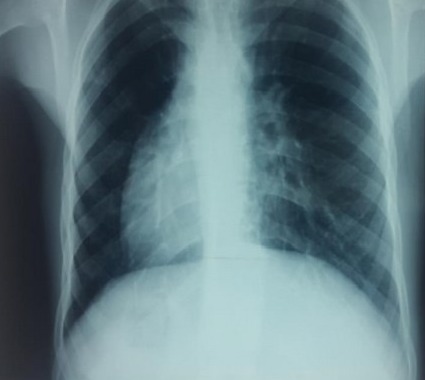
radio thorax réalisée chez notre patient: pointe du cœur à droite, surélévation de la coupole diaphragmatique gauche en rapport avec la présence du foie à gauche

Le diagnostic de lithiase vésiculaire symptomatique sur SI complet était retenu, d’où l’indication d’un traitement chirurgical sous cœlioscopie. Un bilan préanesthésique était établi dont une radio thorax ayant montré une pointe du cœur à droite avec élévation de la coupole diaphragmatique gauche en rapport avec la présence du foie à gauche (Figure 3); un électrocardiogramme (ECG) ayant objectivé une onde P négative en DI et une onde R diminué de V1 à V6 avec un rythme sinusal; une échographie Doppler cardiaque ayant montré un ventricule unique de bonne fonction contractile avec deux valves auriculo-ventriculaires étanches.

L’installation du patient était comme pour une cholécystectomie laparoscopique standard (American position). Le premier trocart de 10mm était introduit sous contrôle visuel au niveau de l’ombilic (*open coelio*) pour l’optique. Un deuxième trocart de 10mm était introduit au niveau de l'épigastre puis deux autres trocarts de 5mm respectivement au niveau de l’hypochondre gauche et au niveau pararectal gauche pour la pince à préhension vésiculaire ainsi que la disposition des trocarts selon le principe de triangulation.

L’exploration montrait que le foie siégeait au niveau de l’hypochondre gauche confirmant ainsi le diagnostic de *situs inversus*. La vésicule biliaire était lithiasique à paroi fine et la voie biliaire principale était de calibre normal. Une dissection du trépied cystique était réalisée à la main droite au crochet coagulateur; tandis que le premier aide maintient le collet de la vésicule biliaire; pour éviter le croisement des bras. Mise en place d’un double clampage proximal et clampage distal puis section du canal cystique, clampage de l'artère cystique et sa section. Enfin, nous avons procédé à la cholécystectomie proprement dite. La vésicule biliaire était extraite d’un sac à travers l'orifice opérateur. Les suites postopératoires étaient simples et l’examen anatomopathologique n'a pas objectivé de signes de malignité.

## Discussion

Le *situs inversus* (SI) est une malformation congénitale rare, de transmission autosomique récessive. Elle est caractérisée par une transposition des organes abdominaux et/ou thoraciques, réalisant une image anatomique en miroir. D’étiologie inconnue, elle a été rapportée pour la première fois par Fabricius en 1600 [1]. Son incidence varie entre 1/10.000 et 1/20.000 naissances selon Mayo *et al.* [2]. Cette malformation peut s'associer à d'autres affections entrant dans le cadre de syndromes tel celui de Kartagener. Mais jusqu’à ce jour, il n’y a pas eu de preuve quant à la prédisposition des patients porteurs de SI à l’apparition de calculs biliaires. Cependant, ces patients présentent le même risque par rapport à la population générale. Par ailleurs, le premier cas de cholécystectomie laparoscopique sur SI a été rapporté par Campos et Sipes en 1991 [3]. Par la suite 41 autres cas ont été rapportés dans la littérature, et aucune contre-indication à la procédure n’a été citée [4]. Or la présentation de la maladie est différente, vu la localisation inversée de la douleur chez ces patients. Elle peut siéger au niveau de l’épigastre, hypochondre gauche et parfois même l’hypochondre droit [1]. Ainsi, Selon Rao *et al.*, 30% des patients porteurs de *situs inversus* et de lithiase vésiculaire présentent des douleurs épigastriques. La même constatation a été faite par McKay, Blake et Eisenberg, tandis que 10% de ces patients présentent des douleurs de l’hypochondre droit. Dans le reste des cas, la douleur est rapportée au niveau de l’hypochondre gauche ou de l’épigastre [4]. En raison de la localisation inhabituelle de la douleur dans le cas de lithiase vésiculaire chez ces patients, un retard diagnostique peut s’observer. Dans ce cas, c'est l'examen cardiaque et la radiographie du thorax qui orientent vers le diagnostic en montrant une dextrocardie avec une poche à air gastrique située au niveau de l’hypochondre droit.

**Figure 4:**
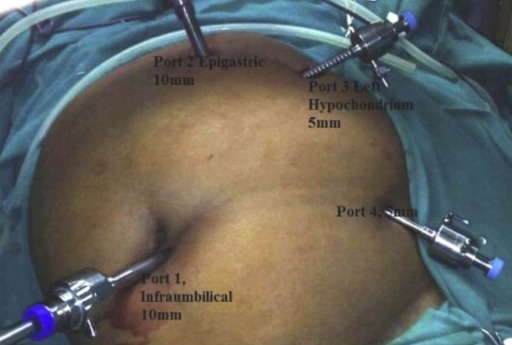
aspect en miroir des orifices des trocarts [1]

**Figure 5:**
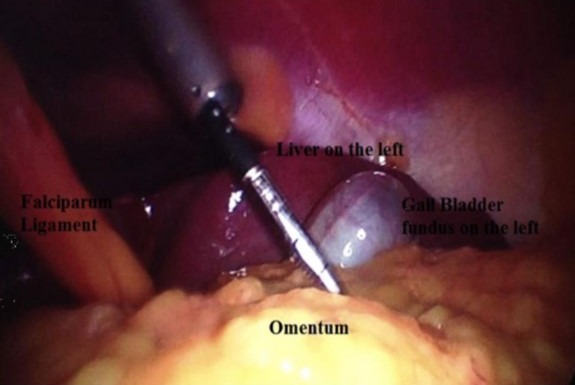
aspect peropératoire montrant le SI [1]

Chez notre patient le diagnostic de SI était déjà connu et il avait présenté des douleurs de l’hypochondre gauche évoquant des coliques hépatiques. Par ailleurs, l’échographie abdominale permet de faire le diagnostic du *situs inversus* en visualisant le foie et la vésicule biliaire au niveau de l’hypochondre gauche et de rechercher la lithiase vésiculaire. Elle représente l’examen de référence (sensibilité et spécificité: 97% Consensus ANAES) [5]. La prise en charge anesthésique est plus délicate chez les patients porteurs de SI, vu l’existence de malformations pulmonaires, vasculaires et surtout cardiaques associées en dehors de la dextrocardie [6, 7]. D’où l'importance du dépistage préopératoire des dysfonctions pulmonaires et cardiaques [6, 8]. La cholécystectomie laparoscopique est l’intervention la plus courante actuellement pour le traitement de la lithiase vésiculaire. Mais elle est techniquement difficile chez les patients porteurs de SI mais n’est pas contre-indiquée. L’image anatomique en miroir nécessite en plus des compétences chirurgicales une planification préopératoire minutieuse de la salle d’opération, de l’équipe chirurgicale, des orifices et des instruments destinés à l’acte. Les difficultés chirurgicales se voient essentiellement chez l’opérateur droitier, qui doit prendre soin de ne pas croiser les bras lors du retrait de la poche d’Hartmann (ou infundibulum vésiculaire) au cours de la dissection du triangle de Calot. Pour procéder à la laparoscopie dans ce cas, l’équipement opératoire est placé du côté gauche du malade. Celui-ci est en position de Trendelenburg inversée avec une légère inclinaison du côté droit. Le pneumopéritoine est créé au niveau du côté gauche, le premier trocart est introduit à travers l’orifice sous ombilical et confirme le SI (Figure 4, Figure 5) [1].

La disposition de l’équipe chirurgicale: l’opérateur principal et le premier aide opératoire se placent du côté droit du patient, le deuxième aide opératoire se place du côté gauche. Un second orifice de 10mm est créé à 5cm sous l’appendice xiphoïde à gauche de la ligne médiane. Les deux autres orifices de 5mm sont créés de la même manière mais du côté gauche. Une pince dentée est introduite à travers le quatrième orifice, utilisée pour le retrait de l’*infundibulum* vésiculaire.

**Figure 6:**
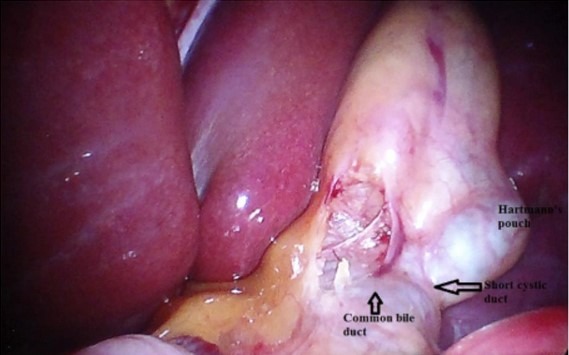
triangle de Calot [1]

Cependant, le premier défi du chirurgien droitier est de retirer la poche d’Hartmann au cours de la dissection du triangle de Calot ce qui conduit à un croisement des mains (Figure 6). Cette difficulté opératoire est surmontée en permettant au premier aide de la retirer tandis que l’opérateur principal dissèque le triangle de Calot de sa main droite à travers l’orifice épigastrique [1]. Le canal cystique est disséqué séparément de l’artère cystique et les deux sont coupés et divisés à travers l’orifice épigastrique. Il peut être jugé difficile d’appliquer les clips quand l’angle de l’applicateur de clips ne correspond pas à l’angle de direction de l’artère cystique. La vésicule biliaire (VB) est disséquée au niveau de la fosse vésiculaire à l’aide du bistouri électrique à travers l’orifice épigastrique, tandis que le premier aide maintient en traction la VB. Lors du retrait de la VB, les calculs sont récupérés à l’aide de la pince ovale et l’organe est retiré à travers l’orifice épigastrique [1].

La durée de l’intervention est estimée à 95 minutes, ce qui est long par rapport à la cholécystectomie conventionnelle, vu les modifications ergonomiques conçues pour s’adapter à l’image en miroir [1]. Ainsi, l’organisation de l’équipe chirurgicale, la mise en place des orifices de trocarts pour la dissection du triangle de Calot et l’application des clips selon le sens du canal et de l’artère cystique sont les éléments les plus importants à considérer lors de la cholécystectomie laparoscopique chez les patients porteurs de SI. La plupart des données de la littérature rapportent la disposition en miroir de l’équipe chirurgicale, des orifices de trocarts et de la salle opératoire [9, 10, 11]. Batista *et al.* ont décrit la procédure avec 4 orifices: pararectal droit de 10mm, ombilical de 10mm, pararectal gauche de 5mm et épigastrique [12].

## Conclusion

La cholécystectomie laparoscopique pour lithiase vésiculaire symptomatique, en cas de *situs inversus* est une intervention originale du fait de la vision en miroir des organes intrapéritonéaux. La voie coelioscopique est faisable moyennant une dissection prudente.

## Conflits d’intérêts

Les auteurs ne déclarent aucun conflit d’intérêts.
